# Arrangements for the Coming Meeting Texas State Medical Assc.

**Published:** 1887-03

**Authors:** 


### SOCIETY NOTES.

### ARRANGEMENTS FOR THE COMING MEETING T. S. M. A., APRIL 26.

The general committee of arrangements consisting of the entire local profession who are members of the Travis County Medical Society, have appointed all the necessary sub-committees, and active preparations are being made to insure a pleasant and successful meeting. The programme has not been perfected, but we are authorized to announce that the entertainment will consist the first night, of a grand reception of members and their ladies, at the Driskill Hotel, with music. The ladies of Austin will be invited to assist in receiving our guests—(no refreshments). (The proprietors of the Hotel also very generously place at the disposal of the Association its grand parlors for committee meetings, etc.) Second evening, a reception at the University, where it is proposed to entertain visitors by electrical or chemical experiments and demonstrations; and a reception also at the Blind Institute. Third evening, reception at the Governor's. Fourth evening, President's address; after which a grand banquet to members, will close the sessions. Addresses of welcome will, of course, be delivered at the hall on opening, by representatives of the profession and of the laity, and by the Mayor. It will not be possible to announce the programme of exercises till chairmen and secretaries of Sections have been heard from. In our April number will be published the programme complete in every detail. Programmes will also be struck off for use of members.

The committee on transportation have secured two-third rates: i. e., members pay two cents per mile each way. This is a reduction of 33⅓ per cent. In view of the fact that for several years past the Roads have refused the medical delegates any discount, this is very handsome, and will be appreciated. The committee hope yet to obtain further reduction on some of the roads. The committee on hotels, &c., report a liberal discount, as follows: Driskill Hotel, whose rates are four dollars, three dollars a day to delegates and their families; the Avenue, Brunswick, Orr, Carrollton, Austin, Raymond and Granbury Hotels each $1.50 a day. A large number of first class boarding houses will take medical delegates and their families at one dollar a day. A delegation from the Committee of Reception will meet guests on arrival of each train, and will furnish them with cards containing the name and location of each hotel and boarding house, together with rates to delegates and families. The Committee on Halls has not yet reported, but it is expected that the capitol building will be secured, and the hall of representatives used for general sessions, and the Senate and lobbies for exhibits, etc., while numerous rooms will be provided for sections.

The following are the committees appointed :

Entertainment, Dr. J. W. McLaughlin, Chairman.
Reception, Dr. F. E. Daniel, Chairman.

Transportation, Dr. T. D. Wooten, Chairman.
Programme, Dr. R. M. Swearingen, Chairman.
Finance, Dr. J. J. Tobin, Chairman.
Music and Halls, Dr. Frank Rainey, Chairman.
Hotel Rates, &c., Dr. Q. C. Smith, Chairman.

Each Chairman has selected his own associates.

For information, members and physicians who contemplate taking the journey, can address any of the members of the general committee.

DANIEL'S TEXAS MEDICAL JOURNAL will issue a daily edition gratuitously.

